# Adherence of HIV clinics to guidelines for the delivery of TB screening among people living with HIV/AIDS in Ghana

**DOI:** 10.1186/s12913-021-07121-9

**Published:** 2021-10-16

**Authors:** Solomon A. Narh-Bana, Tobias F. Chirwa, Esnat D. Chirwa, Frank Bonsu, Latifat Ibisomi, Mary Kawonga

**Affiliations:** 1 School of Public Health, Faculty of Health Sciences, University of the Witwatersrand, Johannesburg, South Africa; 2 Dodowa Health Research Centre, Research and Development Division, Ghana Health Service, Dodowa, Ghana; 3 Gender & Health Research Unit, Medical Research Council, Johannesburg, South Africa; 4 National TB Control Programme, Accra, Ghana; 5 Nigerian Institute of Medical Research, Yaba, Lagos Nigeria; 6 Department of Community Health, Charlotte Maxeke Johannesburg Academic Hospital, Johannesburg, South Africa

**Keywords:** Programme adherence, Facility characteristics, TB screening, PLHIV, HIV clinics, Implementation research, Ghana

## Abstract

**Background:**

Tuberculosis screening of people living with HIV (PLHIV) – an intervention to reduce the burden of TB among PLHIV – is being implemented at HIV clinics in Ghana since 2007, but TB screening coverage remains low. Facility adherence to intervention guidelines may be a factor but is missing in implementation science literature. This study assesses the level of HIV clinic adherence to the guidelines and related facility characteristics in selected district hospitals in Ghana.

**Methods:**

This cross-sectional study was conducted in all 27 district hospitals with HIV clinics, X-ray and geneXpert machines in Ghana. These hospitals are in 27 districts representing about 27% of the 100 district hospitals with HIV clinics in Ghana. A data collection tool with 18-items (maximum score of 29) was developed from the TB/HIV collaborative guidelines to assess facility adherence to four interrelated components of the TB screening programme as stated in the guidelines: intensive TB case-finding among PLHIV (ITCF), Isoniazid preventive therapy initiation (IPT), TB infection control (TIC), and programme review meetings (PRM). Data were collected through record review and interviews with 27 key informants from each hospital. Adherence scores per component were summed to determine an overall adherence score per facility and summarized using medians and converted to proportions. Facility characteristics were assessed and compared across facilities with high (above median) versus low (below median) overall adherence scores, using nonparametric test statistics.

**Results:**

From the 27 key interviews and facility records reviewed, the median adherence scores for ITCF, IPT, TIC, and PRM components were 85.7% (IQR: 85.5–100.0), 0% (IQR: 0–66.7), 33.3% (IQR: 33.3–50.0), and 90.0% (IQR: 70.0–90.0), respectively. The overall median adherence score was 62.1% (IQR: 58.6–65.1), and 17 clinics (63%) with overall adherence score above the median were categorized as high adherence. Compared to low adherence facilities, high adherence facilities had statistically significant lower PLHIV clinic attendees per month (256 (IQR: 60–904) vs. 900 (IQR: 609–2622); *p* = 0.042), and lower HIV provider workloads (28.6 (IQR: 8.6–113) vs. 90 (IQR: 66.7–263.5); *p* = 0.046), and most had screening guidelines (76%, *p* < 0.01) and questionnaire (80%, *p* < 0.01) available on-site.

**Conclusion:**

PRM had highest score while the IPT component had the lowest score. Almost a third of the facilities implemented the TB screening programme activities with a high level of adherence to the guidelines. We suggest to ensure adherence to all four components, reducing staff workloads and making TB screening questionnaires and guidelines available on-site would increase facility adherence to the intervention and ultimately achieve intervention targets.

**Supplementary Information:**

The online version contains supplementary material available at 10.1186/s12913-021-07121-9.

## Background

Tuberculosis (TB) is a significant public health problem of global concern [1, 2]. Though curable, it is a significant contributor to deaths among people living with HIV (PLHIV) [3]. Due to their immune-compromised systems, PLHIV are 20–30 times more likely to contract active TB than non-HIV infected people [4, 5]. Among PLHIV with TB, the lifetime risk of dying is 50% compared to 5–10% in non-HIV infected people with TB [6–8].

To reduce the dual burden of TB and HIV in the population, the World Health Organization (WHO) instituted policy guidelines on TB/HIV collaborative activities in service delivery [4, 5]. These WHO policy guidelines [4] recommend the use of a simplified TB screening questionnaire that asks PLHIV about the absence or presence of four clinical TB symptoms (current cough, weight loss, fever and night sweats) in order to identify screen-negative people and initiate them on Isoniazid preventive therapy (IPT) as prophylactic treatment [4, 9, 10]. The screening is to be done at the initial diagnosis or presentation of HIV and every subsequent visit for HIV care [11]. Many countries where the burden of TB/HIV co-infection is high, including Ghana, are implementing TB screening of PLHIV amongst other TB/HIV collaborative interventions [12].

The estimated prevalence of TB in Ghana in 2017 was 152 per 100,000 population, and 27% of TB patients were co-infected with HIV [13]. Ghana started implementing the TB screening among PLHIV in 2007 as a control measure. In 2014 the policy and guidelines for the implementation of TB/HIV collaborative activities in Ghana were revised to provide a detailed way for enhancing the collaboration between the Ghana National Tuberculosis Control Programme (NTP) and the National AIDS/STI Control Programme (NACP) [5]. The revised document [5] outlined responsibilities for all levels of delivery in TB/HIV collaboration, including the health facility and healthcare providers level. With the aim of reducing TB burden among PLHIV, the revised policy set out targets for TB screening coverage (56%, 64%, 70%, 80%, 85% and 90% in 2015 to 2020 respectively) among PLHIV in HIV care or treatment settings [5, 14]. The TB screening intervention as per the NTP is ongoing at all district hospitals (DHs) with a functioning HIV clinic, as set out in the detailed policy and guidelines [5].

Evidence from the literature [7, 9, 15, 16] shows that TB screening among PLHIV is a critical and necessary step to implementing Isoniazid preventive therapy (IPT) initiation, towards reducing the burden of TB in PLHIV. However, the expected outcome of the intervention has not been fully achieved in Ghana since its implementation. For instance, about 80 and 41% of PLHIV attending clinics in Ghana were not screened for TB in 2013 and 2014 respectively [5, 17] (this was part of the reason for the 2014 TB/HIV policy review) and 47.9% in 2019 [18]. What is not clear is why this intervention of proven effectiveness [19] is not producing the expected result in Ghana. Implementation effectiveness is determined by measuring implementation outcomes such as implementation fidelity [20], sometimes referred to as adherence, which is defined as implementing an intervention according to programme design or guideline [21].

Health programs often provide implementation guidelines for *(1)* the organization/facility (programme level guidelines to which health facilities or other implementing units should adhere), and *(2)* healthcare providers to apply (clinical guidelines) [4, 5, 22–24]. The extent of adherence to TB screening among PLHIV at the organizational or facility level is an essential discussion missing in the implementation science literature. Furthermore, research shows that factors such as *organizational structure*, *staffing availability*, *physical facilities and resources*, and *geographical location* influence adherence to health programs [25–28], no research is available on the extent to which health facilities in Ghana adhere to the programme guideline on TB screening of PLHIV.

This study aim was to investigate programme fidelity or the level of adherence by district hospital HIV clinics in Ghana to the guidelines on the delivery of TB screening amongst PLHIV. In the context of our study, programme adherence refers to the degree to which the DHs deliver the TB screening of PLHIV intervention according to the Ghana NTP guideline. However, the extent to which DHs in Ghana adhere to these programme guideline components has not been assessed. We expected that the level of adherence by the health facilities to the guidelines would vary across facility characteristics. Therefore our secondary aim was to examine the significant differences in facility characteristics between facilities with high adherence and low adherence level. We anticipate the result can inform the designers of the NTP and the TB screening programme at the facility level on areas for improvement in the implementation process.

## Methods

### Study setting

Ghana is administratively divided into 216 districts across 10 regions [29]. These regions are further grouped into three ecological zones (Savannah, Forest and Coastal zones) based on their economic activities and ecology. The health system is divided along the existing administrative regions and districts [30], with decentralized health management teams at the district level. The DHs serve as the first point of referrals from the peripheral health facilities and provide both out-patient and in-patient services. Each DH provides services to a population of 100,000 to 200,000 people with between 50 to 60 available beds. Each district is expected to have a DH, but as of 2017, there were 144 DHs across the country [30], 100 of which had an HIV clinic or treatment setting, and delivered the TB screening intervention targeting PLHIV attending the HIV clinic [5].

A team of health professionals led by the head of the district hospital (medical doctor) facilitates the delivery of the TB/HIV collaborative activities at the district hospital level. The head of the facility is the point of call for undertaking any health-facility based intervention. Other key team members include the facility TB/HIV coordinator (disease control officer or nurse) appointed to be responsible for running the day-to-day implementation and oversight of the TB/HIV collaborative activities and head of the HIV clinic (medical assistant) who manages staff and activities at the clinic.

The NTP guideline outlines the activities required at all the HIV clinics for the delivery of TB screening of PLHIV [5, 14]. These activities include ensuring the availability of an HIV clinic, providing services for routine TB screening for PLHIV, providing TB confirmatory procedures through sputum testing or chest x-ray, ensuring TB infection and control activities, and conducting regular meetings on TB case finding with HIV care providers for feedback [4, 5]. The use of IPT in all PLHIV was not a national policy in Ghana; however, it is supported in HIV specialized cases and children under 5 years where patients are supervised to go through and complete treatment [5]. In 2017, the Ghana NTP earmarked 27 of the 100 DHs with HIV care or treatment services to implement the initiation of IPT for all PLHIV who screened negative on TB screening [31]. According to the IPT implementation plan by the Ghana Health Service, the 27 facilities were selected because they were ART sites with both digital x-ray machines and GeneXpert at the time of implementation. The justification was that it is easier to monitor them as initial roll-out sites because they had molecular diagnostic equipment such the digital x-ray and GeneXpert machines needed to rule out active TB before initiating the IPT. This study was therefore conducted in these 27 DHs representing 27% of the 100 DHs with HIV clinics in Ghana.

### Study design

This was a cross-sectional and census-based study which is part of a more extensive study that assessed the implementation fidelity and determinants of TB screening and IPT initiation among HIV clients attending HIV clinics in Ghana. Every evidence-based intervention has critical components [32–34], and how well these components are implemented determines the effectiveness or success of the intervention [35]. According to Carroll et al.’s conceptualization of fidelity [21], adherence is defined in terms of four constructs – content (implementing the required intervention content), frequency, duration and coverage.

To investigate the level of adherence by the DHs to the intervention guideline, we adapted the Conceptual Framework for Implementation Fidelity proposed by Carroll [21]. Three of Carroll’s four constructs – content, frequency and coverage of the intervention [21] – were thought applicable and relevant to this study.

### Components of the intervention

Intervention implementation in a health facility follows a “top-down” approach (facility level to the provider level) [36]; adherence at the facility level (“top”) influences the degree to which an intervention is implemented [21] at the provider level (“bottom”).

From the content of the NTP guidelines (which also align with WHO’s recommendation), we identified four components of the intervention as follows:

1. *Intensive TB case-finding among PLHIV (ITCF)* through the HIV clinic by screening all PLHIV attending HIV clinic for TB, conducting TB evaluation and the extent to which there was regular or routine delivery of these activities as required by the guidelines;
2. *IPT initiation (IPT)* in specialized cases recommended by clinicians. These include children under 5 years who are exposed to people with active TB and persons with immunosuppression as a result of chemotherapy, prolonged steroid use and persons on renal replacement therapy [5];
3. *TB infection control (TIC)* to prevent the spread and increase of TB in the facility through activities such as having TB infection control plan, a mandatory examination of all health workers for TB and administrative, environmental and personal protection control; and
4. *Programme review meeting (PRM)* to be conducted monthly to involve all the HIV clinic staff discussing issues with quality data sharing and feedback.

These components and their activities were designed to align with NTP guidelines. The four major components identified from the guideline comprised some activities that when implemented, the aim of the intervention will be realized.

### Study sample

All the 27 DHs that had been earmarked by the NTP to be the first facilities providing IPT initiation for PLHIV were included in this study. The NTP listed those 27 facilities to implement the integrated TB/HIV intervention (IPT initiation) based on the fact that they have functioning HIV clinics and have both digital X-ray and GeneXpert testing capacity to rule out TB disease [37].

For this study, the intent was to interview the 27 DHs heads, but where the head was unavailable, we interviewed the facility TB/HIV coordinator or the head of the HIV clinic instead. The TB registers were reviewed and data extracted for the year 2018. The assessment of fidelity in this study was from the perspective of the hospital managers.

### Data collection and measurement

Data collection was from 9th –24th April 2019 by a team of 12 field officers. Using the intervention components identified, we developed an adherence assessment tool because none existed. The tool which was partly questionnaire and partly data extraction form was designed to score the extent to which the DHs adhered to the guidelines – i.e. whether they undertook the prescribed activities of the four components (ITCF, IPT, TIC, and PRM). These activities were therefore used as variables on the tool. Data were collected in two stages on weekdays. The first stage was a face-to-face interview through the administration of a semi-structured questionnaire to the head of the DH or appointed representative. The questionnaire was structured to collect data on the socio-demographic (age sex, cadre, etc.) of the respondent, TB screening activities provided by the facility, and availability of resources (staff, TB screening questionnaire, TB/HIV clinical manual, etc.). The second stage involved using a predesigned data extraction form to extract from the TB and HIV clinic care registers monthly aggregated TB patient data for the period of 1 January to 31December 2018. Data on number of new and existing HIV patients, number screened for TB, number presumed TB, number confirmed and number initiated on IPT were extracted. (See Additional file 1.docx which is a table (Table S1) providing detail information on the elements in the data extraction form and the questionnaire). Where any of these data were missing, the health information officer in charge at the HIV clinic was consulted for rectification. Unresolved missing records were classified incomplete and therefore excluded; 2 records were excluded. All the data collection tools were pre-tested in two similar facilities to minimize measurement errors and identify problem areas for rectification. Three interviews were conducted in each facility (Heads of facility, HIV clinic and facility TB/HIV coordinator) and monthly data for 12 months was extracted for each facility.

To measure programme adherence, the four critical components of the intervention (as described above) were identified and the study collected data to measure the facility adherence to each of these components (activities). Adherence was scored per activity. The fact that the guidelines detailed how these activities should be implemented, we scored the activity as adherent/not adherent if the recommended activity was implemented/not implemented accordingly [38]. Table 1 presents the four components of the intervention for TB screening among PLHIV, the items (activities) and score per component, and the source of information.

**Table 1:** TB screening activities and scores by component and source of information

Components of TB screening intervention	Intervention activity	Subcategory of adherence applicable	Activity score (maximum)	Source of information
Intensive TB case-finding among PLHIV (ITCF) (HIV clinic performs these activities)	1. TB screening for PLHIV	Content	1	Interview
	2. Sputum evaluation of presumed TB cases	Content	1	Interview
	3. X-ray evaluation of presumed TB cases	Content	1	Interview
	4. Frequency of TB screening provision at the clinic	Frequency	3	Interview
	5. Screening coverage: proportion of PLHIV screened for TB	Coverage	1	TB and HIV register
IPT initiation (IPT) (HIV clinic initiates IPT to these clients)	6. Specialized cases	Content	2	Interview
	7. Children under 5 years	Content	2	Interview
	8. All PLHIV	Content	2	Interview
TB Infection control (TIC) (DHs provides these activities)	9. General TIC at the facility	Content	2	Interview
	10. Administrative measure	Content	1	Interview
	11. Environmental measure	Content	1	Interview
	12. Personal protective measure	Content	1	Interview
	13. Surveillance of TB among workers	Content	1	Interview
Programme review meeting (PRM) (HIV clinic performs or provide these activities)	14. TB and HIV review meeting	Content	1	Interview
	15. Uniform and quality data	Content	1	Interview
	16. Share and analyses data at all levels	Content	1	Interview
	17. Provide feedback to all levels	Content	1	Interview
	18. HIV clinic holds regular review meeting	Frequency	4	Interview
	19. Review meetings involve all HIV care providers	Coverage	2	Interview
Total	**19**		**29**	

As shown in Table 1, each of the four major components of the intervention comprise a different number of activities (ITCF 5; IPT 3: TIC 5; and PRM 6). Most of the activities were scored 0 (activity not implemented by the DH) or 1 (activity implemented by the DH). Other activities had a score ranging from 1 to 3. For instance, the scores for “frequency of TB screening” ranged from 1 (HIV clinic provides screening for TB on 1 day a week) to 3 (HIV clinic provides screening for TB on every day of the week) while the scores for “review meeting involve all HIV care providers” ranged from 0 (review meeting never include all HIV healthcare providers) to 2 (review meeting always includes all HIV healthcare providers). Respondents were also asked an open-ended question “how facility ensures TB infection control”. The responses were post-coded into three activities (administrative, environmental and personal protective measures and surveillance of TB among workers) with scores of 0 (activity not implemented) or 1 (activity implemented). The total maximum possible adherence score from all the 19 activities was 29 (ITCF 7; IPT 6: TIC 6; and PRM 10) per DH (Table 1).

To examine differences in the facility characteristics between adherence levels, we collected data on the *geographical location* of the DH which is related to the physical location where the DH is situated in the country described by ecological zone (region and zone); *healthcare staffing level and workload* being the aspect of HIV healthcare providers and work distributions in the facility needed to facilitate the success of the implementation (these factors included the *numbers of HIV healthcare providers, PLHIV attending the HIV clinic per month, screened for TB per month, and calculated workload)*; and *physical facilities and resources* consisted of the aspect of inputs available to facilitate the success of the implementation, such as availability of TB screening questionnaire, TB screening guideline, TB IE&C materials and guidelines for infection control in the DH.

### Data management and analysis

The data collected with the paper-based questionnaire were captured onto EpiData version 3.1 software while the service records data were captured in MS Excel. All the 27 facilities involved in the study were assigned 4-letter codes which were used on both datasets, with no identifiers. Both datasets were password-protected and imported into STATA 15 software [39] for cleaning and analysis.

We conducted baseline descriptive analysis of the characteristics of the respondents and the DHs. Descriptive statistics including percentages, ratios, median and interquartile ranges (IQR) were used to summarize the characteristics such as age, sex, profession, DH location, length of time working at the facility, education, and HIV healthcare providers workloads.

We determined adherence score for each of the four intervention components per DH by summing the activity (item) scores for each intervention component. Reliability check was conducted using Cronbach’s alpha for each of the four components of the TB screening programme at the facility level. An equally weighted additive method was further used by summing the four component adherence scores to generate an overall programme or facility adherence score per DH. These adherence scores were further expressed as percentages of the respective maximum possible scores for descriptive analysis. Because there is no known predefined cut-off point to define high or low programme adherence level, a median percentage score was calculated and used as a cut-off to categorise scores into high or low adherence. A percentage score below the calculated median percentage score of 62.1% was categorized as low adherence. This categorization was used to determine the programme adherence level for each facility, whether it fell within the low or high adherence category. The health facilities were described as high or low using proportion. A Mann-Whitney U test, Spearman’s correlation coefficient and Kruskal-Wallis tests at a 5% significance level were used to assess for statistically significant differences in facility characteristics between facilities with overall high adherence score and those with low score.

### Ethical considerations

This study was approved by the University of the Witwatersrand, South Africa (ref: M190110) and the Ethical Review Committee of the Ghana Health Service (ref: GHS-ERC002/01/19). All procedures involving human participants performed in this study were in accordance with the ethical standards of the institutional and national research committee and with the 1964 Helsinki Declaration and its later amendments or comparable ethical standards.

## Results

### Characteristics of the study participants

The study participants therefore consisted of 27 respondents, of which 18 (66.7%) were heads of facilities, 8 (29.7%) were facility TB/HIV coordinators, and 1 (3.7%) was the head of HIV clinic. We found from the study that the mean age of the respondents was 41.2 (standard deviation (SD) = 8.7) and about half of the respondents (*n* = 14, 51.9%) had worked in the facility for less than 5 years. Most of the respondents were males (*n* = 22, 81.5%), had worked in their current position for less than 5 years and (*n* = 15, 55.6%), were physicians (*n* = 22, 81.2%), and had attained postgraduate as the highest level of education (*n* = 21, 77.8%) as shown in Table 2.

**Table 2:** Characteristics of the study participants

Characteristics of respondents	Response Category	Number (%)
Role at the DH	Head of HIV clinic	1 (3.7)
	TB/HIV coordinator	8 (29.6)
	Head of the facility	18 (66.7)
Age in completed years (Mean & SD)		41.2 (8.7)
Gender	Female	5 (18.5)
	Male	22 (81.5)
Number of years working at the facility	< 5 years	14 (51.9)
	**5 years plus**	13 (48.1)
Number years working in current role	< 5 years	15 (55.6)
	**5 years plus**	12 (44.4)
Main occupation of respondent	Non-clinician	5 (18.5)
	Clinician	22 (81.5)
Highest educational level	Post-secondary non-tertiary	6 (22.2)
	Tertiary	21 (77.8)
Facility location (zone)	Savannah	7 (26.0)
	Forest	11 (40.7)
	Coastal	9 (33.3)

*Note*: *DH* District Hospital, *SD* Standard Deviation

### Characteristics of the health facilities

Out of the 27 DHs studied, eleven were geographically located in the Forest zone, nine from Coastal and seven from the Savannah zones. All these DHs had HIV clinics that had adopted the integration of the TB screening intervention among the PLHIV attending the clinic. All the clinics operated five working days per week (excluding public holidays) and 8 h a day from 8 O’clock in the morning with all providers certified by professional bodies accredited by Ghana Health Service. Table 3 provides a descriptive overview of the facilities included in the analysis. The number of HIV healthcare providers per facility ranged from 8 to 10. The monthly HIV clients attendance per facility vary with a median attendance of 609 (IQR: 182–1479) while the monthly median number of PLHIV screened for TB was 79 (IQR:32–344). The median workload (HIV healthcare providers to HIV clinic attendance was 67 (IQR: 18–127). Most (74.1 and 77.8%) of the facilities have TB screening questionnaires and guidelines respectively available. However, other resources such TB/HIV clinical manual, TB information, education and communication (IEC) materials and TB prevention and infection control guidelines as were not available on most facilities. See Additional file 2.docx which is a table (Table S2) providing descriptive information on the characteristics of the facilities by the HIV clinics.

**Table 3:** Characteristics of the health facilities by ecological zones

Variables		Ecological zone Median (IQR), N (%)			*p*-value
	All (*N* = 27)	Savannah (*n* = 7)	Forest (*n* = 11)	Coastal (*n* = 9)	
Median number of HIV healthcare providers per HIV clinic.	9 (8–10)	8 (7–8)	9 (7–11)	10.0 (10–10)	**0.018**
Median number of PLHIV attending the HIV clinic per month.	609 (182–1479)	600 (60–1023)	904 (257–4342)	475 (60–900)	0.177
Median number of PLHIV screened for TB per month.	79 (32–344)	69 (57–419)	200 (58–904)	32 (26–79)	0.313
Median number of patients per provider per monthly	67 (18–127)	66 (8–128)	113 (28–620)	43 (7–90)	0.157
TB screening questionnaire available					0.295
No	7 (25.9)	1 (14.3)	2 (18.2)	4 (44.4)	
Yes	20 (74.1)	6 (85.7)	9 (81,8)	5 (55.6)	
TB/HIV clinical manual available					0.187
No	23 (83.2)	5 (71.4)	11 (100.0)	7 (77.8)	
Yes	4 (14.8)	2 (28.6)	0 (0.0)	2 (22.2)	
TB screening Guideline available					0.606
No	6 (22.2)	1 (14.3)	2 (18.2)	3 (42.7)	
Yes	21 (77.8)	6 (85.7)	9 (81,8)	6 (66.7)	
TB IE&C materials available					0.276
No	24 (88.9)	6 (85.7)	11 (45.8)	7 (77.8)	
Yes	3 (11.1)	1 (14.3)	0 (0.0)	2 (22.2)	
TB prevention and infection control guideline available					0.187
No	23 (83.2)	5 (71.4)	11 (45.8)	7 (77.8)	
Yes	4 (14.8)	2 (28.6)	0 (0.0)	2 (22.2)	

*Note*: *IE&C* Information, education and communication

### TB screening implementation fidelity

#### Adherence scores

**Fig. 1:**
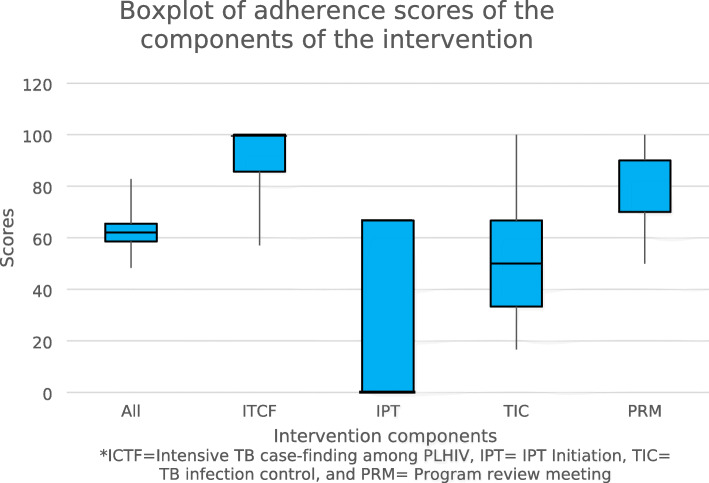
Boxplot showing the distribution of adherence scores of the TB screening intervention components

The overall adherence score and scores for each of the four components of the TB screening of PLHIV intervention are presented by the boxplot in Fig. 1.

The overall adherence score (summation of items of all components) for the facilities ranged from 48.3 to 82.8%, with a median of 62.1% (IQR: 58.6–65.1). The median scores for each of the four components differ. Median scores and IQR for the four components were: ITCF (85.7%; IQR: 85.7, 100%); IPT (0; IQR: 0, 66.7%); TIC (50%; IQR: 33.3, 66.7%); and PRM (90%; 70, 90%). Among the four components, PRM had highest score while the IPT component had the lowest score. Also, with the ITCF component, the 1st quartile and the median scores were the same (85.7%) while the 3rd quartile and the maximum scores were also the same (100%). With the IPT component, the minimum score, the 1st quartile and the median were the same (0%) while the 3rd quartile and the maximum score were the same (66.7%). Similarly, with the PRM component, the median score and the 3rd quartile score was the same (90%). None of the components and the overall had an outlier score.

The internal consistency for each of the four intervention components (ITCF, IPT, TIC, and PRM) and the overall programme were 0.59, 0.94, 0.63, 0.57, and 0.69, respectively. As the calculated alpha approaches one, it indicates that the items measure the components as intended to well and are interrelated within the test [40, 41].

#### Facility/programme adherence level

**Fig. 2:**
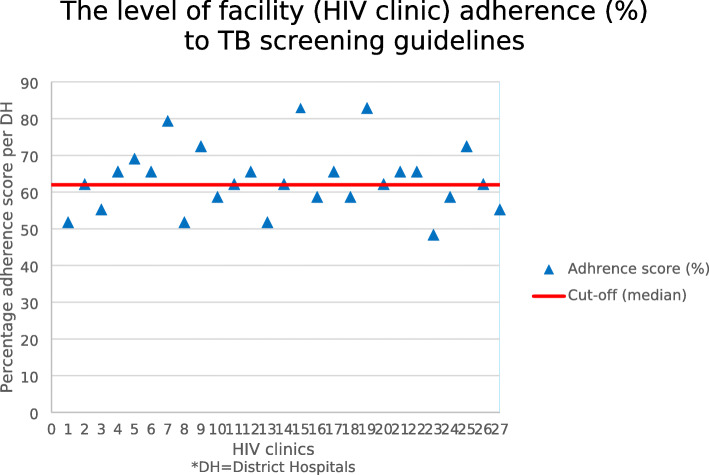
Graphical representation of the facility adherence score (%) to TB screening guidelines and activities at 27 HIV clinics

The programme adherence median score of 62.1% was used as the cut-off point to categorize the facilities into to a low or high adherence level. We found that 17 (63.0%) facilities scored ≥62.1% to fall in the high adherence category, while 10 (37.0%) facilities scored < 62.1% to fall in the low adherence level category as showed by Fig. 2.

### Differences in facility characteristics between high and low adherence level facilities

The characteristics of the facilities studied were grouped as geographical location, Health facility utilization and staffing workload, and physical facilities and resources. In the Table 4, the assessment for significant differences in facility-level factors between the low and high adherence level facilities is presented.

**Table 4:** Association between facility-level factors and programme adherence level

Facility factors	Low adherence level N (%), Median (IQR)	High adherence level N (%), Median (IQR)	Significance
*Geographical location*			
Ecological zone			*p = 0.876
Savannah	2 (28.6)	5 (71.4)	
Forest	5 (45.5)	6 (54.5)	
Coastal	3 (33.3)	6 (66.7)	
*Physical facilities and resources*			
TB screening questionnaire available			**^*p*** **= 0.003**
No	6 (85.7)	1 (14.3)	
Yes	4 (20.0)	16 (80.0)	
TB/HIV clinical manual available			^*p* = 0.370
No	9 (39.1)	14 (60.9)	
Yes	1 (25.0)	3 (75.0)	
TB screening guideline available			**^*p*** **= 0.009**
No	5 (83.3)	1 (16.7)	
Yes	5 (23.8)	16 (76.2)	
TB IE&C materials available			^*p* = 0.503
No	9 (37.5)	15 (62.5)	
Yes	1 (33.3)	2 (66.7)	
TB prevention and infection control guideline available			^*p* = 0.370
`1No	9 (39.1)	14 (60.9)	
Yes	1 (25.0)	3 (75.0)	
*Health facility utilization and staffing workload*			
Median number of HIV healthcare providers per HIV clinic.	9.5 (8–10)	9 (8–10)	^α^*p* = 0.726
Median number of PLHIV attending the HIV clinic per month.	900 (609–2622)	256 (60–904)	^α^***p*** **= 0.042**
Median number of PLHIV screened for TB per month.	74 (26–277)	200 (38–419)	^α^*p* = 0.345
Median number of patients per provider per month	90 (66.7–263.5)	28.6 (8.6–113)	^α^***p*** **= 0.046**

*Note*: * used Kruskal-Wallis test, ^ used Mann-Whitney U, and ^α^ used Spearman’s correlation coefficient to assess significance difference. *IE&C* Information, education and communication

The study shows that most DHs in the Savannah (71.4%), Coastal (66.7%) and Forest (54.6%) zones adhered to the implementation guidelines and activities (high adherence level); however, the result showed that the mean adherence score was not statistically insignificant (*p* = 0.876) between the three groups.

The findings showed that amongst the health facility utilization and staffing workload factors, the mean overall adherence score was statistically significant different in monthly patient load and provider workload between low and high adherence levels. Compared to the high adherence facilities, the low adherence facilities had a significantly higher median number of PLHIV attending HIV clinics per month (900; IQR: 609–2622 vs. 256; IQR: 60–904), and a significantly higher patient to provider ratio (*p* = 0.046). The mean overall adherence score was not statistically significant different (*p* > 0.05) for number of HIV healthcare providers or the number of PLHIV screened for TB at the facilities.

Among the physical facilities and resources factors, the study revealed that only the availability of TB screening questionnaire and TB screening guideline were significantly associated with adherence level. Most HIV clinics where TB screening questionnaires (80.0%) and TB screening guidelines (76.2%) were available, were in the high adherence group while most clinics without these documents for implementing the intervention were in the low adherence group (*p* < 0.01). We found that the mean overall adherence score was not statistically significant different in the availability of TB/HIV clinical manual (*p* = 0.589), TB IE&C materials (*p* = 0.888), and TB prevention and infection control guidelines (*p* = 0.589) between adherence levels.

## Discussion

This study used a cross-sectional design to investigate the adherence level to programme guidelines for implementing the TB screening activities at the HIV clinics and related facility factors at selected HIV clinics in Ghana. Our study found seventeen (63.0%) of the 27 facilities had a high adherence level meaning they implemented most of the activities in the clinic according to the intervention guidelines. The intervention component with the highest adherence score was programme review meeting (PRM) followed by intensive TB case-finding among PLHIV (ITCF) and then TB infection control (TIC) components while the IPT component scored the least among the four components. Although over half the number of DHs studied implement the TB screening intervention with high level adherence to the guideline, the difference in the proportion is suboptimal. The low adherence scores for TIC and especially IPT by some HIV clinics could explain why about 37% of the facilities were in the low programme adherence level. Similarly, Fagan J A, 1990 found in their Violent Juvenile Offender Program that intervention sites that received moderate to high adherence scores or ratings in implementing most of the core components of the program fared much better compared to sites that received weak to moderate scores [42]. Contextual adaptation by modifying what may be adaptable and maintaining the critical components with their respective items may be needed to enhance adherence to TIC and IPT components [43, 44]. Nonetheless, actual reasons for the low adherence to TIC and IPT which would inform scale-up, scale-out and process evaluation needs further research.

The WHO recognizes that implementation guidelines promote standardization and simplification in implementing an effective intervention [45, 46] in real life. But, suboptimal facility adherence level to intervention guidelines is known to result in an unsuccessful intervention outcome [47, 48]. Thus, the finding of this study provides a possible explanation for the low and fluctuating TB screening coverage among PLHIV attending HIV clinics in Ghana, an observation that led to the revision of the TB/HIV collaborative guideline with newly set targets in 2014 [5]. With the facility adherence level found in our study, it will be quite challenging to achieve the 2018 target of 80% TB screening coverage among PLHIV at the HIV clinic in Ghana. No previous study on facility adherence or fidelity on TB screening guidelines available.

Adherence as a determinant of failure or success of the implementation of an intervention is influenced by important factors [49] of which some are related to the organization in which the intervention is being implemented. Given the relationship between facility or organizational factors and how an intervention is implemented in a health facility [25], we examined facility-level factors that were relevant and likely to have an impact on the implementation of the intervention in several studies, including geographical location, staffing and workload, and facility resources [49–53]. Some of these factors examined appeared to have little influence on the adherence level. For example, the relationship between geographical location (being in Savannah, Forest or Coastal zones of Ghana) and programme adherence level was not statistically significant. Notwithstanding, we found some variability between geographical location on level of adherence in the implementation of the TB screening intervention, with facilitates in the Savannah zone having higher adherence compared to the Coastal and Forest zones in that order. In terms of HIV prevalence, the Ghana Demographic and Health Survey report (2014) showed that, most regions in the Savannah zones have the lowest prevalence rates, followed by Coastal zone while the Forest zone has the region with the highest rate [54]. This corroborates our findings that, the Forest zone has the highest median number of PLHIV attending the HIV clinics per month [55, 56]. The variability in adherence levels observed across geographical zones could be due to fewer number of PLHIV attending HIV clinics in Savannah than Forest zones per month. However, our findings may be a function of the small number of sample (27 facilities) involved in the study and insufficient power to conduct robust statistical analyses to test for association. Nonetheless, our findings are consistent with other studies [57, 58].

We also explored the relationship between adherence level and other facility characteristics covering healthcare staffing levels and workloads. We found that facilities with high programme adherence level had significantly lower numbers of PLHIV attending the HIV clinic per month and lower HIV healthcare provider-HIV client ratios than facilities with low programme adherence level. Although there is no significant difference in the number of HIV healthcare provider between the adherence levels, inversely, high adherence level facilities screened more PLHIV for TB than low adherence level facilities; however, the difference is not significant. Facilities seeing more clients screened less PLHIV for TB. Systematic reviews [59, 60] confirmed earlier assertions that provider staffing is associated with intervention outcomes. The provider-patient ratio and the caseload were found to be influencers of implementation outcomes [56, 61] such as fidelity. These findings corroborate our results. Our study further shows that most HIV clinics without the TB screening questionnaire on-site had low level of adherence adhere to the guidelines for implementing the intervention. This finding is consistent with the Global Fund 2018 report on best practices on TB case finding and treatment which indicated that the absence of TB screening questionnaire at the point of service delivery is a possible hindrance to screening PLHIV, TB cases identification and TB outcomes improvement [62]. Availability of guidelines promotes a step-by-step process to a successful implementation of health intervention [63]. Van de Glind et al. (2015) report in their multi-case study that availability of protocols, procedures and agreements supporting the implementation of the intervention influences adherence [64]. However, our study found that other resources such as availability of TB/HIV clinical manual, TB IE&C manual and TB prevention and infection control guideline do not vary with the programme adherence levels.

### Limitation

It is worth mentioning that albeit some promising findings in this study, it also has some limitations to be considered in the interpretation of the results. Firstly, due to our selection procedure, respondents were mostly proponents’ heads of the facility. On the other hand, because of the role and experience in implementing the intervention, respondents were able to provide rather detailed answers to our questions. Secondly, a limitation of concern is the external validity of the findings. The study was conducted in 27 district hospitals, and it is unclear to what extent these findings can be generalized to other district hospitals in Ghana. All the same, it was a census-based survey of all the facilities that met our inclusion criteria. We interpret these data with caution. Also, the median as a cut-off to determine high or low adherence facility does not take into account the magnitude or the distribution of the score across the samples, but because the 27 facility adherence scores were not normally distributed the non-parametric test conducted was appropriate. Thirdly, it should be noted that this study did not investigate the patients’ perspective. It would be valuable to add their contribution to the factors found in this study. Finally our calculated Cronbach’s alpha were generally low. However, the components (ITCF and PRM) with low alpha (0.59 and 0.57 respectively) had a higher adherence score compared to the components (IPT and TIC) with high alpha (0.94 and 0.63 respectively). Although alpha is widely used, the low level observed in this study might either be due to low number of items measuring the specific component or probably not a good measure of internal consistency or reliability for the ITCF and PRM components in our study.

## Conclusion

A majority of the district hospitals implemented the intervention guidelines as is. Adherence was higher for intensive case-finding among PLHIV and programme review meeting intervention components and lower for IPT initiation and TB infection control intervention components. The programme adherence level observed by the DHs may explain the low and fluctuating intervention outcomes observed over the past years. Evidence from our study confirmed an association between programme adherence level and facility characteristics related to *health facility utilization and staffing workload,* and *physical facilities and resources*.

We recommend that NTP must highlight and re-emphasize the importance of implementing the activities of these components fully through meetings, trainings and engagements. This could improve adherence for IPT initiation and TB infection control intervention components. Given the lack of programme adherence level studies, our study adds to implementation science literature a way of assessing adherence level by health facilities implementing an intervention.

## Supplementary Information

**Additional file 1.**

**Additional file 2.**

## Data Availability

The data on the facility level adherence to TB screening among PLHIV is accessible through supplementary material uploaded under supplementary file (filename: programmeuse.dta). **“**Additional file 1” also uploaded under supplementary file (filename: additional file 1.docx) is a supplementary table providing descriptive information on the characteristics of the facility. Additional file 1” also uploaded under supplementary file (filename: additional file 1.docx) is a supplementary table providing descriptive information on the characteristics of the facility.

## Abbreviations

DHs: District hospitals
HIV: Human Immuno virus
IPT: Isoniazid preventive therapy
IQR: Interquartile range
ITCF: Intensive TB case-finding among PLHIV
NACP: National AIDS/STI Control Programme
NTP: National Tuberculosis Control Programme
PLHIV: People living with HIV
PRM: Programme review meeting
TB: Tuberculosis
TIC: TB infection control
WHO: World Health Organization
