# Supplementary material for: Adherence of HIV clinics to guidelines for the delivery of TB screening among people living with HIV/AIDS in Ghana
Source: BMC Health Serv Res. 2021 Oct 16;21:1110. doi: 10.1186/s12913-021-07121-9 (PMC8520611; doi:10.1186/s12913-021-07121-9)
Supplement: Supplementary file 1 — Additional file 1. [file 12913_2021_7121_MOESM1_ESM.docx]

Table S1: Detail elements on the data extraction form and the structured questionnaire

| **Data extract data form** | | | | | | | | | | | | | |
| --- | --- | --- | --- | --- | --- | --- | --- | --- | --- | --- | --- | --- | --- |
| Month | # Attendants | # On ART | # Screened for TB | **Presumptive TB** | | | | | | **Non Presumptive TB** | | | |
|  |  |  |  | # Presumed TB | # X-ray test | # GeneXpert test | # Confirmed Negative | # Initiate IPT | | # Non Presumed TB | # X-ray test | # Confirmed Negative | # Initiate IPT |
| 1 |  |  |  |  |  |  |  |  | |  |  |  |  |
|  |  |  |  |  |  |  |  |  | |  |  |  |  |
| 12 |  |  |  |  |  |  |  |  | |  |  |  |  |
| **Structured questionnaire** | | | | | | | | | | | | | |
| **Socio-Demographic Information** | | | | | | | | | | | | | |
|  | Respondent | | | | 1. Head of HIV clinic 2. TB/HIV co-ordinator 3. Head of facility | | | | | | | | |
|  | Age (in completed years) | | | |  | | | | | | | | |
|  | Sex | | | | 1. Male 2. Female | | | | | | | | |
|  | Number of years working in this HIV care clinic (in completed years) | | | | | | | |  | | | | |
|  | Occupation category/Cadre of health worker | | | | 1. Doctor 2. Medical Assistant 3. Professional Nurse 4. Auxiliary nurse  5. Health Information officer 6. Other (specify) | | | | | | | | |
|  | Number of years in your current capacity / role | | | |  | | | | | | | | |
|  | Level of education (highest level completed) | | | | 1. Postgraduate degree 2. Bachelor Degree 3. Diploma 4. Certificate 5. Secondary  6. Junior Secondary | | | | | | | | |
| **TB screening activities** | | | | | | | | | | | | | |
|  | Do you provide any of the following TB/HIV services in this HIV care clinic? | | | | **Content**  0. No  1. Yes | | **Frequency**  1. One day a week 2. A few days a week (1-4) 3. Every day of the week (5 day) 4.Other (specify): 88. Not applicable | | | | | | |
|  | TB screening for PLHIV | | | |  | |  | | | | | | |
|  | Sputum testing for PLHIV (any method) | | | |  | |  | | | | | | |
|  | Chest x-ray for TB testing | | | |  | |  | | | | | | |
|  | How do you ensure TB infection control in your clinic? | | | |  | |  | | | | | | |
|  | Does this HIV care clinic provide IPT for PLHIV? | | | | | |  | 0. No 1. Yes | | | | | |
|  | **If yes: To which group/s of clients do you provide IPT?** | | | | | | | | | | | | |
|  | Only Children | | | | 0. No 1. Yes | | How long has this clinic been providing IPT for this group? | | | | | | |
|  | Only Pregnant women | | | | 0. No 1. Yes | | How long has this clinic been providing IPT for this group? | | | | | | |
|  | Specialized cases recommended by clinician | | | | 0. No 1. Yes | | How long has this clinic been providing IPT for this group? | | | | | | |
|  | All PLHIV | | | | 0. No 1. Yes | | How long has this clinic been providing IPT for this group? | | | | | | |
|  | Does this facility conducts meetings on TB case finding or TB screening of PLHIV | | | | 0. Never 2. Annually 3. Quarterly 4. Monthly 5. Other (specify) 6. Weekly | | | | | | | | |
|  | Does the meeting on the TB case finding programme include the HIV care providers | | | | 0. Never 2. Annually 3. Quarterly 4. Monthly 5. Other (specify) 6. Weekly | | | | | | | | |
|  | What is the purpose of these meetings and what is discussed there? | | | | 1. Providing feedback to providers on their practice 2. Sharing of data 3. Discuss TB/HIV training 4. Other (specify)……… | | | | | | | | |
|  | On which days of the week is this facility open? | | | | 1. Daily 2. Once a week 3. Twice a week 4. Thrice a week 5. Four time a week | | | | | | | | |
|  | How do you handle infection control in this HIV care clinic? | | | | 1. Infection control plan 2. Infection control guidelines made available to staff 3. Infection control training for staff | | | | | | | | |
|  | How many health workers have had infection control training in this HIV care clinic? | | | | | | | |  | | | | |
|  | How often does your facility assess for TB infection among health care workers? | | | | 0. Never 2. Annually 3. Quarterly 4. Monthly 5. Other (specify) 6. Weekly | | | | | | | | |
|  | Please may you describe how this facility monitors HIV providers in their provision of TB screening and IPT service? | | | | | | | |  | | | | |
| **Availability of resources** | | | | | | | | | | | | | |
|  | TB screening questionnaire / algorithm | | | | | 0. No 1. Yes | | | | | | | |
|  | TB/HIV clinical manual | | | | | 0. No 1. Yes | | | | | | | |
|  | Guidelines for TB screening | | | | | 0. No 1. Yes | | | | | | | |
|  | Guidelines for IPT provision | | | | | 0. No 1. Yes | | | | | | | |
|  | IEC materials for use by the providers\ | | | | | 0. No 1. Yes | | | | | | | |
|  | Infection control guidelines | | | | | 0. No 1. Yes | | | | | | | |
|  | Does your facility have lab facilities for sputum testing? | | | | | 0. No 1. Yes | | | | | | | |
|  | Number of HIV healthcare providers? | | | | |  | | | | | | | |
