# Supplementary material for: Adherence of HIV clinics to guidelines for the delivery of TB screening among people living with HIV/AIDS in Ghana
Source: BMC Health Serv Res. 2021 Oct 16;21:1110. doi: 10.1186/s12913-021-07121-9 (PMC8520611; doi:10.1186/s12913-021-07121-9)
Supplement: Supplementary file 2 — Additional file 2. [file 12913_2021_7121_MOESM2_ESM.docx]

Table S2: Characteristics of the health facility by HIV clinics and by zone

| **Health facility** | **Number of HIV healthcare providers** | **Average number of PLHIV attending the HIV clinic monthly** | **Average number of PLHIV screened for TB monthly** | **Average number of patients per provider monthly** | **TB screening questionnaire available. Yes/No** | **TB/HIV clinical manual available. Yes/No** | **TB screening Guideline available. Yes/No** | **TB IE&C materials available**  **Yes/No** | **TB prevention and infection control guideline available**  **Yes/No** |
| --- | --- | --- | --- | --- | --- | --- | --- | --- | --- |
| **HIV clinics in the Forest zone** | | | | | | | | | |
| 1 | 12 | 2622 | 174 | 218.5 | Yes | No | Yes | No | No |
| 2 | 9 | 257 | 257 | 28.6 | Yes | No | Yes | No | No |
| 3 | 8 | 904 | 904 | 113 | Yes | No | Yes | No | No |
| 4 | 7 | 345 | 11 | 49.3 | Yes | No | Yes | No | No |
| 5 | 8 | 3634 | 1321 | 454.3 | No | No | No | No | No |
| 6 | 7 | 4342 | 244 | 620.3 | No | No | No | No | No |
| 7 | 7 | 121 | 58 | 17.3 | Yes | No | Yes | No | No |
| 8 | 11 | 201 | 200 | 18.3 | Yes | No | Yes | No | No |
| 9 | 10 | 609 | 177 | 60.9 | Yes | No | Yes | No | No |
| 10 | 9 | 7380 | 7 | 820 | Yes | No | Yes | No | No |
| 11 | 12 | 8792 | 8002 | 732.7 | Yes | No | Yes | No | No |
| **HIV clinics in the Coastal zone** | | | | | | | | | |
| 12 | 10 | 900 | 19 | 90 | No | No | No | No | No |
| 13 | 12 | 1479 | 1479 | 123.3 | Yes | Yes | Yes | Yes | Yes |
| 14 | 10 | 900 | 26 | 90 | No | Yes | Yes | Yes | Yes |
| 15 | 11 | 475 | 344 | 43 | Yes | No | Yes | No | No |
| 16 | 9 | 182 | 25 | 20.2 | Yes | No | Yes | No | No |
| 17 | 10 | 806 | 79 | 80.6 | Yes | No | No | No | No |
| 18 | 8 | 60 | 58 | 7.5 | Yes | No | No | No | No |
| 19 | 10 | 37 | 29 | 3.7 | No | No | Yes | No | No |
| 20 | 10 | 45 | 32 | 4.5 | No | No | Yes | No | No |
| **HIV clinics in the Savannah zone** | | | | | | | | | |
| 21 | 7 | 706 | 704 | 100.9 | Yes | Yes | Yes | No | Yes |
| 22 | 9 | 600 | 69 | 66.7 | Yes | No | Yes | No | No |
| 23 | 8 | 1023 | 308 | 127.9 | Yes | No | Yes | No | No |
| 24 | 7 | 421 | 419 | 60.1 | Yes | No | Yes | No | No |
| 25 | 8 | 38 | 2108 | 60 | Yes | No | Yes | No | No |
| 26 | 8 | 38 | 57 | 60 | No | No | No | No | No |
| 27 | 7 | 4.8 | 263.5 | 8.6 | Yes | No | Yes | Yes | Yes |
| **All** | 9 (8–10) | 609 (182–1479) | 79 (32–344) | 67 (18 – 127) | No=7  Yes=20 | No=23  Yes=4 | No=6  Yes=21 | No=24  Yes=3 | N0=23  Yes=4 |

***Note: IE&C=Information, education and communication***
